# Thulium fiber laser versus holmium:YAG laser for ureteroscopic lithotripsy: a single-center retrospective cohort study

**DOI:** 10.3389/fsurg.2026.1880083

**Published:** 2026-06-17

**Authors:** Longfei Yang, Yangfeng Lou, Ruipeng Li, Junhua Li, Chenhao Tang, Yanbin Wang, Peng Zhou, Chen Song

**Affiliations:** Department of Urology, Hangzhou Third People’s Hospital, Hangzhou, China

**Keywords:** holmium:YAG laser, laser lithotripsy, stone-free rate, thulium fiber laser, ureteral stones

## Abstract

**Objective:**

To evaluate and compare the clinical efficacy of thulium fiber laser (TFL) versus holmium:YAG laser (Ho:YAG) in the treatment of ureteral stones in a retrospective, non-randomized setting.

**Methods:**

We conducted a single-center retrospective cohort study of 132 patients with ureteral stones who underwent ureteroscopic laser lithotripsy between September 2023 to June 2025. Patients were stratified into TFL (*n* = 54) and Ho:YAG (*n* = 60) groups after applying exclusion criteria. Baseline demographics, stone characteristics (size, location, density), and preoperative hydronephrosis were recorded. Primary outcomes included operative time, laser time, and stone-free rate (SFR) assessed by non-contrast computed tomography (NCCT) at 12 weeks. Secondary outcomes encompassed intraoperative stone retropulsion, visual quality, complication rates, and hospital stay.

**Results:**

Baseline characteristics were comparable between groups. The TFL group showed better intraoperative visual quality (*p* = 0.045), shorter operative time (median 23.5 vs. 33.5 min, *p* = 0.001), shorter laser time (median 5 vs. 11.5 min, *p* < 0.001), and less stone retropulsion (*p* = 0.027). Intraoperative complications, including bleeding (7.4% vs. 20.0%, *p* = 0.043) and ureteral injury (1.9% vs. 11.7%, *p* = 0.028), were lower in the TFL group. Hospital stay and overall postoperative complication rates were similar. The 12-week SFR as assessed by NCCT was 96.3% in the TFL group versus 86.7% in the Ho:YAG group (*p* = 0.026).

**Conclusion:**

In this retrospective, non-randomized cohort study, our findings suggest that thulium fiber laser (TFL) may be associated with shorter operative and laser time, better intraoperative visualization, fewer intraoperative complications, and a higher stone-free rate (SFR) confirmed by non-contrast computed tomography (NCCT) at 12 weeks postoperatively. However, limited by the retrospective, non-randomized study design and short follow-up duration, well-designed prospective randomized controlled trials with extended safety surveillance periods are warranted to confirm these findings.

## Introduction

Urinary stone disease is one of the most common urological disorders worldwide. Despite treatment, recurrence is possible, with reported recurrence rates exceeding 50% within 5–10 years, making it a lifelong condition (1). The incidence of urinary stones has been increasing in recent years. Clinical presentations vary, including colicky pain, nausea, vomiting, voiding dysfunction, hematuria, and infection with fever (2). Current treatment modalities for urinary stones include extracorporeal shock wave lithotripsy (ESWL), ureteroscopic laser lithotripsy, percutaneous nephrolithotomy (PCNL), and laparoscopic ureterolithotomy. Ureteroscopic laser lithotripsy is one of the primary approaches for managing urinary stones. In recent years, the widespread adoption of laser lithotripsy and its favorable outcomes, particularly holmium laser lithotripsy, have established it as the gold standard for endoscopic treatment of urinary stones (3).

The super-pulsed thulium fiber laser (TFL) is a novel laser platform characterized by its lightweight, compact size, and high energy conversion efficiency (4). Emitting at a wavelength of 1940nm, which is close to the water absorption peak, its water absorption coefficient is approximately four times that of holmium:YAG laser (Ho:YAG), making it a promising lithotripsy tool (4, 5). *In vitro* studies have shown that the TFL has superior stone ablation efficiency compared to Ho:YAG. Clinical trials have also confirmed that the TFL offers higher lithotripsy efficiency and fewer complications than Ho:YAG for urinary stones (5, 6).

However, the existing randomized controlled trials have predominantly been conducted in Western populations and adopted inconsistent definitions of stone-free rate (SFR), with some relying on kidney-ureter-bladder (KUB) radiography or ultrasound rather than non-contrast computed tomography (NCCT) (7). To date, real-world evidence regarding the SFR confirmed by the more sensitive NCCT in Asian populations remains very limited. This retrospective study aimed to evaluate the clinical efficacy of TFL versus Ho:YAG for ureteral calculi in a Chinese patient population, with SFR assessed by NCCT, and to supplement real-world data to the growing body of comparative literature.

## Materials and methods

### Study design and patients

This single-center retrospective study was approved by the relevant Institutional Review Board. We reviewed patients who underwent ureteroscopic laser lithotripsy for ureteral stones between September 2023 and June 2025.

#### Inclusion criteria

Patients aged 18–80 years with a solitary ureteral stone confirmed on preoperative non-contrast computed tomography (NCCT) and undergoing primary ureteroscopic lithotripsy with either Ho:YAG or TFL.

#### Exclusion criteria

(1) Concurrent urinary tract anomalies, active urinary infection, or malignancy; (2) Coagulopathy or significant hematologic disorders; (3) Unstable cardiovascular or cerebrovascular disease; (4) Pregnancy; (5) Incomplete follow-up data or loss to follow-up before the 12-week postoperative assessment.

### Surgical technique and equipment

All procedures were performed by a single experienced senior urologist, assisted by another urologist, to minimize operator-related variability. General anesthesia was used in all cases.

#### Ho:YAG group

Lithotripsy was performed using a VersaPulse PowerSuite 80/100W dual-wavelength laser (Ho:YAG & Nd:YAG) (Lumenis Ltd., Beijing, China) with pulse energy of 0.5–1.0 J and frequency of 10–15 Hz, yielding an average power of 5–15W. Lithotripsy was performed using a fragmentation technique.

#### TFL group

Lithotripsy was performed using a super-pulsed thulium fiber laser (SRM-T1F, Runke Laser Technology Co., Ltd., Shanghai, China) with two lithotripsy modes: fragmentation mode at pulse energy of 0.5–1.0 J and frequency of 10–15 Hz, and dusting mode at pulse energy of 0.1–0.2 J and frequency of 100 Hz. Lithotripsy was performed using a combination of fragmentation and dusting techniques.

A 200-μm laser fiber was employed in both groups. A double-J ureteral stent was placed at the conclusion of every procedure.

### Outcome measures

Primary outcomes were total operative time (from initial ureteroscope insertion to final double-J stent placement), laser-on time (total duration of laser activation), and the stone-free rate (SFR) at 4 and 12 weeks. SFR was defined as either no residual fragments or only clinically insignificant residual fragments (CIRFs) ≤ 3 mm on NCCT. NCCT images were reviewed independently by a radiologist who was blinded to the laser group assignment.

Secondary outcomes included: intraoperative visual quality (subjectively scored by the primary surgeon using a 3-point scale: 0 = clear/excellent, 1 = moderate/acceptable, 2 = poor/obscured); intraoperative stone retropulsion (graded as: 0 = none, 1 = mild movement within the ureter, 2 = significant migration or escape into the renal pelvis, jointly assessed by the surgeon and assistant); intraoperative complications (bleeding impairing vision, ureteral injury graded by the Traxer classification (8); postoperative complications (e.g., fever >38.0 °C, lower urinary tract infection, gross hematuria, others such as lumbar soreness/pain, bladder irritation), graded using the Clavien-Dindo classification system; and length of hospital stay (days).

### Follow-up

Follow-up was conducted via telephone, WeChat, and outpatient clinic visits. NCCT was performed at 4 and 12 weeks postoperatively.

### Statistical analysis

Statistical analysis was performed using IBM SPSS Statistics version 25.0. Continuous variables are presented as mean ± standard deviation (SD) if normally distributed, or as median with interquartile range (IQR) if non-normally distributed, and were compared using the independent Student's t-test or the Mann–Whitney U test, respectively. Categorical variables are expressed as numbers and percentages and were compared using the Chi-square test or Fisher's exact test, as appropriate. A two-tailed *p*-value of <0.05 was considered statistically significant for all analyses.

## Results

**Figure 1:**
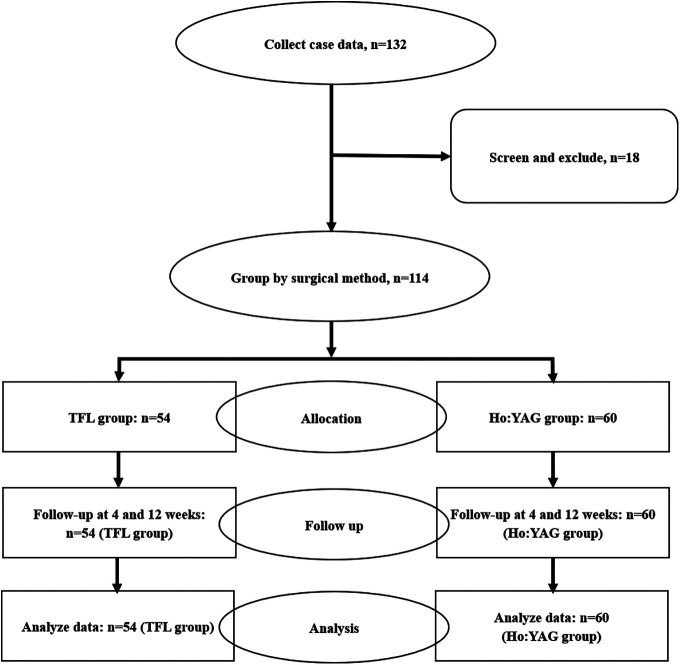
Patient enrollment flow chart.

Figure 1 shows the flow chart of this retrospective study. Of 132 initially collected patients, 18 were excluded based on criteria, resulting in 114 patients for analysis (54 in the TFL group, 60 in the Ho:YAG group). The TFL group comprised 32 males and 22 females, with a median age of 50.5 (IQR 41, 57.5) years. The Ho:YAG group comprised 36 males and 24 females, with a median age of 49.5 (IQR 38.8, 60.2) years. Table 1 details the baseline characteristics including age, sex, stone features, and preoperative hydronephrosis, showing no significant differences between groups, indicating reasonable baseline comparability.

**Table 1:** Baseline clinical characteristics of the patients.

Characteristic	TFL group (*n* = 54)	Ho:YAG group (*n* = 60)	*P*-value
Age, median (IQR), years	50.5 (41,57.5)	49.5 (38.8,60.2)	0.235
Sex, *n* (%)			0.913
Male	32 (59.3)	36 (60.0)	
Female	22 (40.7)	24 (40.0)	
Stone size (max diameter), mean ± SD, cm	1.2 ± 0.4	1.3 ± 0.4	0.157
Stone CT value, median (IQR), Hu	821 (685,923)	800 (631,962)	0.672
Stone location, *n* (%)			0.930
Upper ureter	16 (29.6)	19 (31.7)	
Middle ureter	25 (46.3)	26 (43.3)	
Lower ureter	13 (24.1)	15 (25.0)	
Side, *n* (%)			0.963
Right	34 (63.0)	38 (63.3)	
Left	20 (37.0)	22 (36.7)	
Preoperative hydronephrosis, *n* (%)			0.900
None	19 (35.2)	18 (30.0)	
Mild	24 (44.4)	26 (43.3)	
Moderate	9 (16.7)	13 (21.7)	
Severe	2 (3.7)	3 (5.0)	

SD, standard deviation; IQR, interquartile range; Hu, Hounsfield units.

### Intraoperative outcomes

**Figure 2:**
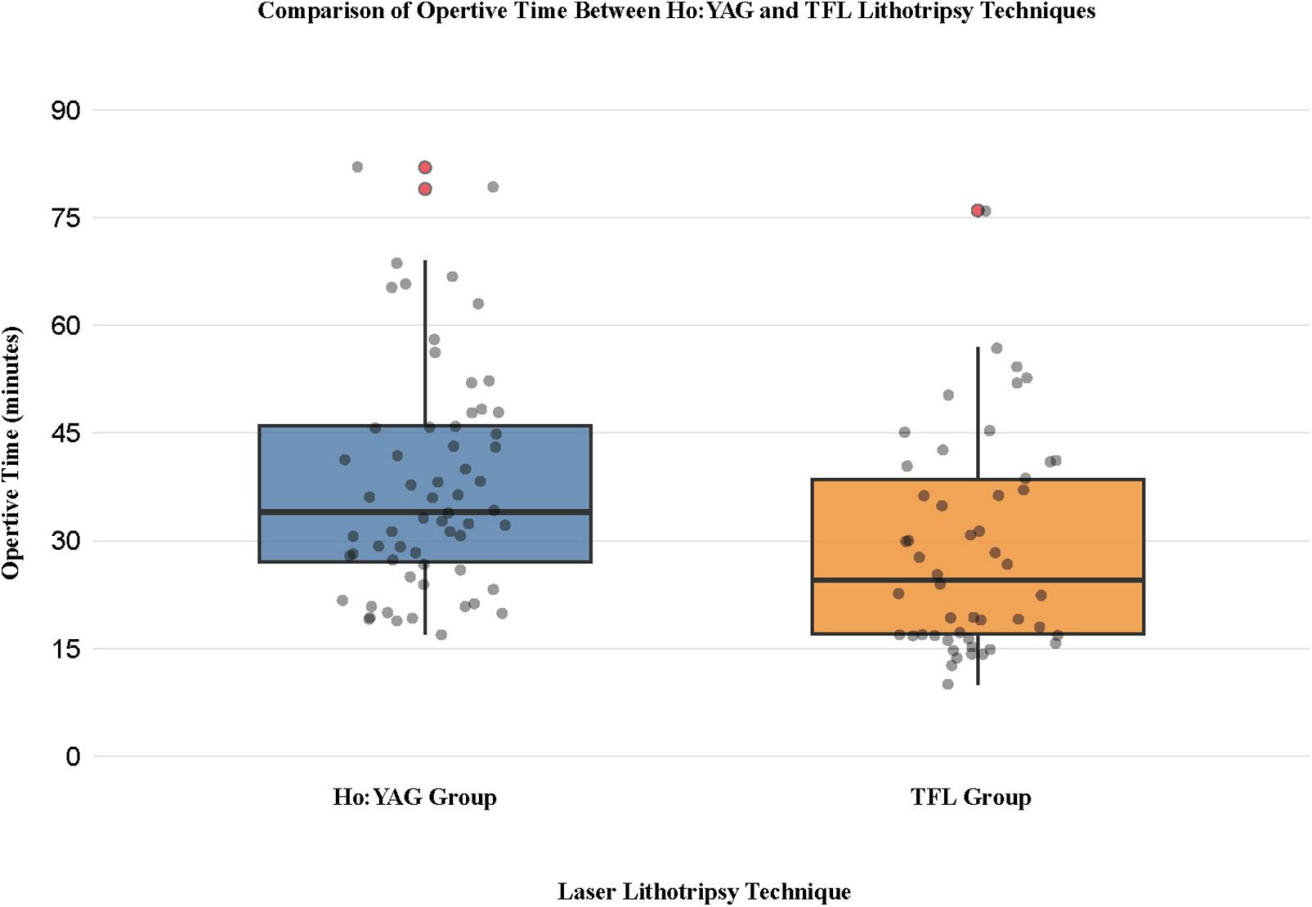
Box plot comparing operative time between the two groups.

**Figure 3:**
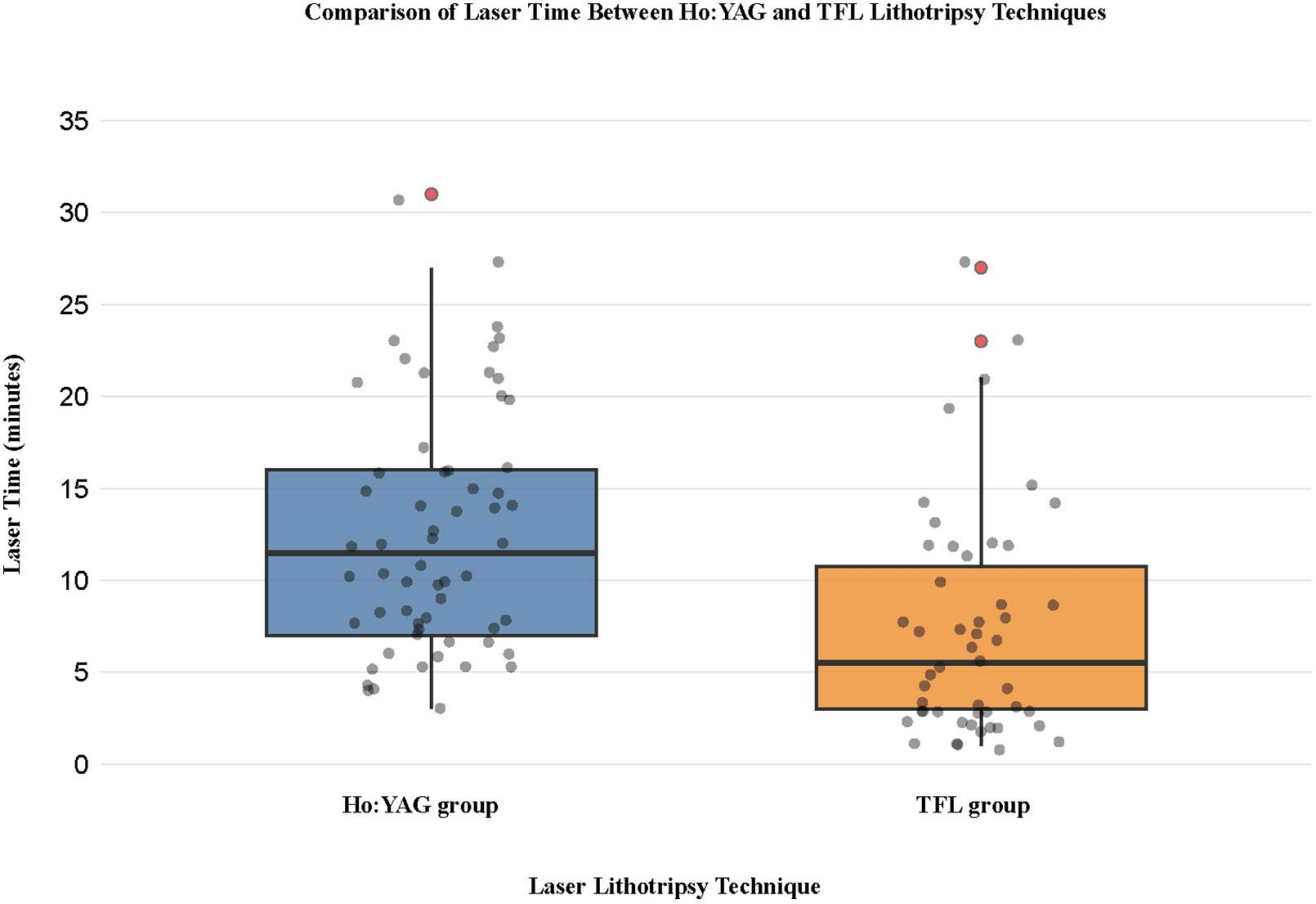
Box plot comparing laser time between the two groups.

Factors affecting the surgical procedure, such as stone retropulsion and intraoperative visibility, were evaluated and scored by two surgeons. The total operative time was significantly shorter in the TFL group compared to the Ho:YAG group (23.5 [17, 38.5] min vs. 33.5 [26.8, 46] min, *p* = 0.001). Additionally, the laser lithotripsy time was markedly shorter in the TFL group (5 [3, 10.8] min vs. 11.5 [7, 16] min, *p* < 0.001). The differences in operative time and laser time can be intuitively visualized from the box plots presented in Figures 2, 3.

During lithotripsy, intraoperative visibility was significantly better in the TFL group (*p* = 0.045). Stone retropulsion was less frequent in the TFL group, with only 2 cases scored as grade 2, whereas the Ho:YAG group had 8 cases with grade 2 retropulsion, showing a statistically significant difference between the groups (*p* = 0.027). In the TFL group, 2 patients with stone retropulsion into the renal pelvis underwent lithotripsy using flexible ureteroscopy. In the Ho:YAG group, 5 patients experienced stone retropulsion into the renal pelvis; 4 were managed with flexible ureteroscopy, while 1 had a ureteral stent placed and underwent a second laser lithotripsy session 2 weeks later. All patients received a double-J ureteral stent, which was removed 2–4 weeks postoperatively. The total hospital stay did not differ significantly between the two groups (2.8 ± 0.9 vs. 3.1 ± 1.0 days, *p* = 0.102). Table 2 summarizes the intraoperative parameters and total hospital stay for both groups.

**Table 2:** Intraoperative parameters and hospital stay.

Parameter	TFL group (*n* = 54)	Ho:YAG group (*n* = 60)	*P-*value
Operative time, median (IQR), min	23.5 (17,38.5)	33.5 (26.8,46)	0.001
Laser time, median (IQR), min	5 (3,10.8)	11.5 (7,16)	<0.001
Intraoperative visibility, *n* (%)			0.045
Score 0	46 (85.2)	38 (63.3)	
Score 1	5 (9.2)	15 (25.0)	
Score 2	3 (5.6)	7 (11.7)	
Stone retropulsion, *n* (%)			0.027
Score 0	48 (88.9)	43 (71.7)	
Score 1	4 (7.4)	9 (15.0)	
Score 2	2 (3.7)	8 (13.3)	
Hospital stay, mean ± SD, days	2.8 ± 0.9	3.1 ± 1.0	0.102

SD, standard deviation; IQR, interquartile range.

### Complications

Ureteral injuries were graded according to the Traxer and Thomas classification system (8). The TFL group had 1 ureteral injury and 4 bleeding events; the Ho:YAG group had 7 ureteral injuries (4 perforations, 3 mucosal tears) and 12 bleeding events, with significant differences (*p* < 0.05). Pus was noted above the stone in 2 TFL and 3 Ho:YAG patients; only one Ho:YAG patient developed postoperative fever, which cured with antibiotics. Postoperative complications in the TFL group included 2 lower UTIs, 12 cases of gross hematuria, 3 fever episodes, and 10 other symptoms (lumbar discomfort, bladder irritation). The Ho:YAG group had 4 lower UTIs, 16 gross hematuria cases, 5 fever episodes, and 13 other symptoms, with no significant difference between groups (*p* = 0.090). One patient was readmitted for a perinephric hematoma managed conservatively in the Ho:YAG group. All postoperative complications were Clavien–Dindo grade I or II. Table 3 details intra- and postoperative complications.

**Table 3:** Comparison of intra- and postoperative complications.

Complication	TFL group (*n* = 54)	Ho:YAG group (*n* = 60)	*P-*value
Intraoperative complications^a^, *n* (%)			
Bleeding (Clavien I)	4 (7.4)	12 (20.0)	0.043
Ureteral injury			0.028
Mucosal perforation (Clavien I) (Clavien I)	0 (0.0)	4 (6.7)	
Mucosal tear (Clavien II)	1 (1.9)	3 (5)	
Postoperative complications^a^, *n* (%)			0.090
Fever (>38.0 °C) (Clavien II)	3 (5.6)	5 (8.3)	
Lower UTI (Clavien I)	2 (3.7)	4 (6.7)	
Perinephric hematoma (Clavien II)	0 (0.00)	1 (1.7)	
Gross hematuria (Clavien I)	12 (22.2)	16 (26.7)	
Other (lumbar discomfort/pain/bladder irritation) (Clavien I/II)	10 (18.5)	13 (21.7)	

Clavien, Clavien–Dindo classification system.

a The Clavien–Dindo classification system is a standardized tool for assessing the severity of surgical complications. It categorizes complications into five grades (from Grade I to Grade V) based on the complexity of interventions required to manage them, with higher grades indicating more severe complications.

### Stone-free rate and hydronephrosis

NCCT was performed at 4 and 12 weeks postoperatively. The 4-week SFR did not differ significantly (88.9% vs. 83.3%, *p* = 0.364). At 12 weeks, the SFR was significantly higher in the TFL group (96.3% vs. 86.7%, *p* = 0.026). Hydronephrosis improvement rates were similar at 12 weeks (*p* = 0.846). Table 4 presents the SFR and hydronephrosis outcomes.

**Table 4:** Stone-free rate at 4 and 12 weeks and hydronephrosis postoperatively.

Outcomes	TFL group (*n* = 54)	Ho:YAG group (*n* = 60)	*P*-value
4-week SFR, % (*n*)	88.9 (48/54)	83.3 (50/60)	0.364
12-week SFR, % (*n*)	96.3 (52/54)	86.7 (52/60)	0.026
Hydronephrosis^a^, *n* (%)			0.846
None	41 (75.9)	42 (70.0)	
Mild	9 (16.6)	11 (18.4)	
Moderate	3 (5.6)	5 (8.3)	
Severe	1 (1.9)	2 (3.3)	

a hydronephrosis at 12 weeks postoperatively.

## Discussion

Since its introduction, laser technology has been widely applied across various urological diseases. The Ho:YAG, with its reliable performance, high lithotripsy efficiency, and applicability to diverse stone types, has been adopted globally as the gold standard for surgical lithotripsy. However, it has drawbacks such as significant stone retropulsion and migration. Hence, new laser technologies are needed to mitigate these issues. The TFL, as a new generation device for urinary stone treatment, has shown promising results in clinical practice. *In vitro* studies indicate that under equivalent energy settings, the TFL reduces stone retropulsion and achieves ablation rates up to four times higher than the Ho:YAG (4, 9).

This retrospective study compared the efficacy of the TFL and the Ho:YAG lithotripsy, with follow-up NCCT at 4 and 12 weeks. At 4 weeks, SFR was similar between groups. However, at 12 weeks, SFR was significantly higher in the TFL group. Laser settings were similar in both groups. Laser time was significantly shorter in the TFL group, and intraoperative visibility was rated as better by the surgeons. These findings are broadly consistent with previous clinical studies, but should be interpreted as exploratory given the subjective nature of visibility and retropulsion assessments. Ulvik et al. (5) conducted a prospective randomized controlled trial including 120 patients and found that for ureteral stones, both lasers achieved 100% SFR at 12 weeks, but the TFL resulted in shorter laser time, fewer intraoperative complications, higher fragmentation efficiency, and similar postoperative complications.

Laser fiber diameter is one factor influencing lithotripsy efficacy. the Ho:YAG typically requires larger-core fibers, whereas TFL can operate efficiently with smaller fibers (e.g., 150 μm, 100 μm, even 50 μm), which allow better irrigation flow and consequently better surgical visibility (10). In our study, a 200-μm fiber was used for both groups.

Stone retropulsion also affects lithotripsy outcomes. In our study, stone retropulsion was jointly assessed by the surgeon and assistant. Stone retropulsion occurred in 6 the TFL group cases and 17 the Ho:YAG group cases, being significantly less frequent with the TFL. This difference may be attributed to distinct laser-tissue interaction mechanisms: the Ho:YAG relies on photothermal and photomechanical effects, while the TFL primarily utilizes photothermal ablation with weaker mechanical effects (11). Furthermore, the Ho:YAG has a higher peak power and uneven energy distribution, generating larger vapor bubbles that promote stone movement (12). In contrast, the TFL has a lower peak power and more uniform energy distribution, producing smaller bubbles and less stone displacement (12–14) Blackmon et al. (15) reported less stone retropulsion with the TFL compared to the Ho:YAG. A single-center prospective randomized trial by Haas et al. (7) also demonstrated significantly lower stone retropulsion with the TFL, enhancing its lithotripsy efficiency. Clinical studies by Enikeev (16), Ventimiglia (12), and others have confirmed that, at comparable energy, frequency, and average power, the TFL causes less stone retropulsion, provides higher fragmentation efficiency, and offers better visual clarity than the Ho:YAG. Clear visual field is crucial for efficient lithotripsy, facilitating manipulation, reducing operative time, potentially lowering infection risks associated with prolonged surgery, and minimizing ureteral trauma (17). Our findings of better visibility with the TFL in this cohort align with these reports.

Ureteral injuries of varying degrees occurred in both groups. Mucosal tears or perforations are generally related to surgical technique. Except for one patient in the Ho:YAG group who required ureteral stent placement due to stone migration and underwent a second laser lithotripsy session two weeks later, no other patients had their operations terminated due to intraoperative complications. However, intraoperative bleeding impairing visibility was observed more frequently in the Ho:YAG group. Ulvik et al. (5) also reported higher intraoperative bleeding with the Ho:YAG, attributing the lower bleeding rate with the TFL to its higher water absorption coefficient and lower tissue ablation threshold. Overall operative time was significantly shorter with the TFL in this study, consistent with findings by Alexey (6) and Ulvik (5), likely due to better visibility and less stone retropulsion.

Post-laser lithotripsy complications may include infection, fever, lumbar discomfort, pain, gross hematuria, and bladder irritation. The Clavien–Dindo classification provides a standardized assessment of surgical complication severity. In our series, postoperative complications in both groups were all Clavien–Dindo grade I or II, with no significant difference between groups (*p* = 0.090). One patient developed symptomatic perinephric hematoma after discharge, managed conservatively with good recovery in the Ho:YAG group; follow-up NCCT at 3 months showed significant hematoma resolution.

Several limitations of our study must be acknowledged. First, its retrospective, non-randomized design may introduce selection bias and unmeasured confounders, therefore, causal relationships between laser type and outcomes cannot be established. Second, although the sample size was adequate to detect differences in primary endpoints, it may be underpowered for subgroup analyses or rare adverse events. Third, the lack of detailed stone composition data prevents analysis of laser efficacy across different stone types. Fourth, the intraoperative assessments of visual quality and stone retropulsion were subjective, rated by the operating surgeons without externally validated standards, and may have fluctuated within a single procedure; consequently, these findings should be considered exploratory. Fifth, while follow-up included outpatient clinic visits, the majority of postoperative contacts were conducted virtually via telephone or WeChat, and no standardized physical examinations were performed, which may deviate from usual clinical practice in some centers. Finally, follow-up was limited to 12 weeks; long-term outcomes regarding stone recurrence and ureteral stricture rates remain to be investigated. Despite these limitations, our findings align with the growing body of evidence supporting the clinical utility of the TFL.

## Conclusion

In this single-center retrospective cohort, our observations suggest that TFL lithotripsy may be associated with shorter operative time, a lower incidence of intraoperative complications, and a higher postoperative stone-free rate. However, due to the retrospective design, small sample size, subjective outcome measures, and lack of external validation, these findings should be interpreted as hypothesis-generating rather than definitive proof of superiority. Given its potential advantages during lithotripsy, TFL remains a promising novel option for the minimally invasive management of urinary stones (18), but large-scale, prospective randomized controlled trials with prolonged follow-up are warranted to validate these observations and assess its cost-effectiveness in clinical practice.

## Data Availability

The original contributions presented in the study are included in the article/Supplementary Material, further inquiries can be directed to the corresponding author.
